# Alpha-enolase as a potential cancer prognostic marker promotes cell growth, migration, and invasion in glioma

**DOI:** 10.1186/1476-4598-13-65

**Published:** 2014-03-21

**Authors:** Ye Song, Qisheng Luo, Hao Long, Zheng Hu, Tianshi Que, Xi’an Zhang, Zhiyong Li, Gang Wang, Liu Yi, Zhen Liu, WeiYi Fang, Songtao Qi

**Affiliations:** 1 Department of Neurosurgery, Nanfang Hospital, Southern Medical University, Guangzhou, Guangdong, PR China; 2 Cancer Research Institute of Southern Medical University, Guangzhou, Guangdong, PR China; 3 Department of Neurosurgery, Affiliated Hospital, Youjiang Medical College for Nationalities, Baise, Guangxi, PR China; 4 Department of Pathology, Basic School of Guangzhou Medical College, Guangzhou, Guangdong, PR China

**Keywords:** ENO1, Glioma, Cell growth, EMT, PI3K/Akt

## Abstract

**Background:**

The success of using glycolytic inhibitors for cancer treatment relies on better understanding the roles of each frequently deregulated glycolytic genes in cancer. This report analyzed the involvement of a key glycolytic enzyme, alpha-enolase (ENO1), in tumor progression and prognosis of human glioma.

**Methods:**

ENO1 expression levels were examined in glioma tissues and normal brain (NB) tissues. The molecular mechanisms of ENO1 expression and its effects on cell growth, migration and invasion were also explored by 3-[4,5-dimethylthiazol-2-yl]-2,5 diphenyl tetrazolium bromide (MTT) assay, Transwell chamber assay, Boyden chamber assay, Western blot and in vivo tumorigenesis in nude mice.

**Results:**

ENO1 mRNA and protein levels were upregulated in glioma tissues compared to NB. In addition, increased ENO1 was associated disease progression in glioma samples. Knocking down ENO1 expression not only significantly decreased cell proliferation, but also markedly inhibited cell migration and invasion as well as in vivo tumorigenesis. Mechanistic analyses revealed that Cyclin D1, Cyclin E1, pRb, and NF-κB were downregulated after stable ENO1 knockdown in glioma U251 and U87 cells. Conversely, knockdown of ENO1 resulted in restoration of E-cadherin expression and suppression of mesenchymal cell markers, such as Vimentin, Snail, N-Cadherin, β-Catenin and Slug. Furthermore, ENO1 suppression inactivated PI3K/Akt pathway regulating the cell growth and epithelial-mesenchymal transition (EMT) progression.

**Conclusion:**

Overexpression of ENO1 is associated with glioma progression. Knockdown of ENO1 expression led to suppressed cell growth, migration and invasion progression by inactivating the PI3K/Akt pathway in glioma cells.

## Background

Malignant gliomas account for the vast majority of adult malignant brain tumors that are graded according to the WHO classification system, which has implications for prognosis and management [1]. The current standard therapy includes maximal safe resection followed by radiotherapy in combination with temozolomide [2]. A majority of patients succumb to the disease within 2 years of diagnosis [3].

Glioblastoma, like most cancers, possesses a unique bioenergetic state of aerobic glycolysis known as the Warburg effect [4]. Malignant glioma cells thrive despite an irregular blood supply and frequently in a hypoxic microenvironment [5]. Compensatory mechanisms, including glucose uptake and glycolytic activity, are increased in these tumors [6]. Recent studies indicated that some glycolytic enzymes are complicated, multifaceted proteins rather than simple components of the glycolytic pathway [7].

Enolases are glycolytic enzymes responsible for the ATP-generated conversion of 2-phosphoglycerate to phosphoenolpyruvate. In mammals, there are three different enolase isoforms: alpha-, beta,-and gamma-enolase. Each are encoded by three distinct genes and expressed in a tissue and development-specific manner [8,9]. The alpha-enolase (ENO1) is a key glycolytic enzyme that plays a functional role in several physiological processes depending on its cellular localization [10]. It mainly localizes in the cytoplasm as a 48 kDa ENO1 enzyme, while an alternatively translated form is predominantly in the nuclear region. The nuclear form has been characterized as a 37 kDa c-Myc promoter-binding protein (MBP-1), and is negative transcription factor [11]. In tumor cells, ENO1 is upregulated and activated by several glucose transporters and glycolytic enzymes that contribute to the Warburg effect [12]. Warburg observed that cancer cells consume more glucose than normal cells and generate ATP by converting pyruvate to lactic acid, even in the presence of a normal oxygen supply. Increased ENO1 gene activity and protein production has been detected in several tumors [13]. ENO1 glycolytic activity is strongly associated with increased ATP citrate lyase expression in gliomas [14], thus ENO1 may act as a metabolic tumor promoter conferring a selective growth advantage onto ENO1-overexpressing tumor cells. The exact mechanisms by which ENO1 expression is mediated and its function in glioma are not well understood currently.

In this study, we evaluated the expression of ENO1 in human patient samples. To explore its associated molecular mechanisms in glioma cells, we examined the effect of targeted silencing of ENO1 gene on cell proliferation, migration and invasion using shRNA in vitro and vivo. These studies will be useful in identifying potential candidates for targeted therapeutic intervention of glioma.

## Results

### Expression of ENO1 gene in glioma and NB tissues

**Figure 1:**
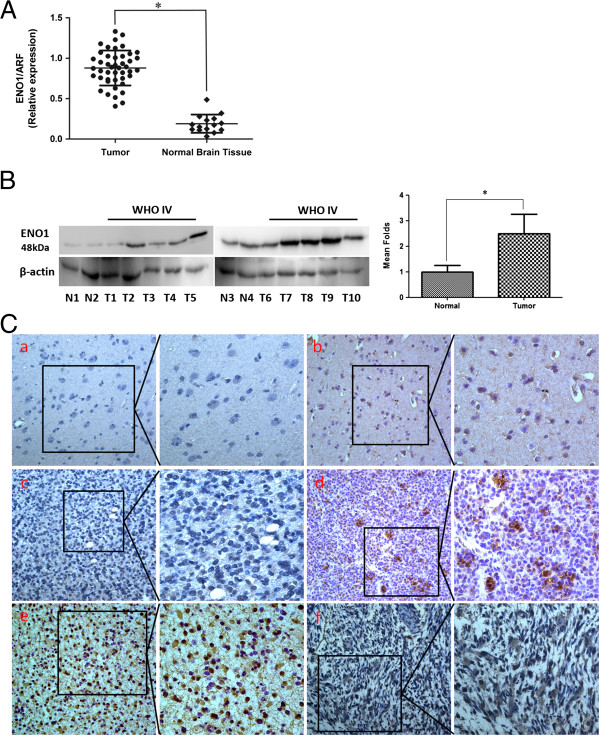
**Expression of ENO1 gene in glioma and NB tissues. (A)**. mRNA expression of ENO1 is decreased in NB tissues compared with glioma tissues by real-time PCR assay. Data are presented were presented as mean ± SD for three independent experiments (*P < 0.05). **(B)**. Western blot analysis of ENO1 expression in 10 cases of WHO IV glioma tissue samples compared with 4 NB tissues. The unpaired t-test was used for this assay (*P < 0.05). **(C)**. ENO1 expression was increased in 136 primary glioma samples compared to 15 normal brain tissues using immunohistochemical staining. **a)**. Weak staining of ENO1 in NB tissues. **b)**. Strong staining of ENO1 in NB tissues. **c** and **d)**. Weak staining of ENO1 in glioma samples. **e** and **f)**. Strong staining of ENO1 in glioma samples. Original magnification 400 × .

In order to assess the role of ENO1 in glioma, we performed real-time PCR to measure the expression of ENO1 mRNA transcripts in 45 freshly collected glioma tissues and 15 freshly collected NB tissues. Compared with NB tissues, glioma tissues exhibited higher expression levels of ENO1 mRNA (*P* < 0.0001) (Figure 1A). ENO1 protein (48 kDa) was found to be up-regulated in 10 cases of glioma (WHO IV) compared with 4 NB tissues by Western blot (*P* < 0.0001) (Figure 1B). We also measured the expression levels and subcellular localization of ENO1 protein in 136 archived paraffin-embedded glioma samples and 15 NB tissues using immunohistochemical staining (Figure 1C). ENO1 protein was highly expressed in 69.1% (94/136) of glioma samples, while only in 20.0% (3/15) of NB samples, a significantly lower frequency (*P* < 0.001) (Table 1).

**Table 1:** Protein expression of HDGF between glioma and NB tissues

**Group**	**Cases**	**Protein expression**		***P *** **value**
		**High expression**	**Low expression**	
Glioma	136	94	42	
Normal	15	4	11	0.000

### Relationship between clinicopathologic characteristics and ENO1 expression in glioma patients

The relationship between clinicopathologic characteristics and ENO1 expression levels in individuals with glioma are summarized in Table 2. We found no significant association between ENO1 expression levels and patients’ age, sex or histologic type in the 136 glioma cases. However, we observed that the expression level of ENO1 was positively correlated with the status of pathology classification (WHO I-II vs. WHO III-IV) (*P* = 0.000) in glioma patients (Table 2). To determine whether ENO1 is an independent prognostic factor for glioma, we performed multivariate analysis of ENO1 expression adjusted for the same parameters. The results indicated that the level of ENO1 expression was an independent prognostic factor for glioma (*P* < 0.001) (Table 3).

**Table 2:** Correlation between the clinicopathologic characteristics and expression of HDGF protein in glioma

**Characteristics**	**n**	**ENO1 (%)**		***P***
		**High-expression**	**Low-expression**	
Gender				
Male	88	63 (71.6%)	25 (28.4%)	
Female	48	31 (64.6%)	17 (35.4%)	0.398
Age				
≥50	73	50 (68.5%)	23 (31.5%)	
<50	63	44 (69.8%)	19 (30.2%)	0.865
Histologic type				
Astrocytic tumors	98	67 (68.4%)	31 (31.6%)	
Oligodendrogial tumors	15	14 (93.3%)	1 (6.7%)	
Oligoastrocytic tumors	23	13 (56.5%)	10 (43.5%)	0.053
WHO grade				
I + II	41	18 (43.9%)	23 (56.1%)	
III + IV	95	76 (80.0%)	19 (20.0%)	0.000

**Table 3:** Summary of univariate and multivariate Cox regression analysis of overall survival duration

	**Univariate analysis**			**Multivariate analysis**		
	**P**	**HR**	**95% CI**	**P**	**HR**	**95% CI**
Age						
<50 vs. ≥ 50 years	0.716	1.069	0.745-1.534	0.989	1.003	0.691-1.456
Gender						
Male vs. female	0.834	1.041	0.716-1.514	0.177	1.303	0.887-1.915
Histologic type						
AT vs. OT vs. OAT^*^	0.849	1.041	0.689-1.574	0.187	1.335	0.870-2.048
ENO1 expression						
Low vs. High	0.000	20.978	9.087-48.430	0.000	26.214	10.766-63.827

^*^AT: Astrocytic tumors; OT: Oligodendrogial tumors; OAT: Oligoastrocytic tumors.

### Survival analysis

**Figure 2:**
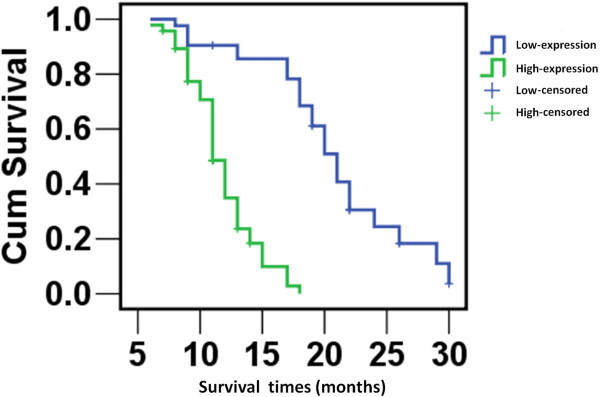
**Kaplan-Meier survival analysis of overall survival duration in 136 glioma patients according to ENO1 protein expression.** Accumulation expression of ENO1 was unfavorable for glioma prognosis. The log-rank test was used to calculate *P* values.

To investigate the prognostic value of ENO1 expression in glioma, we assessed the association between levels of tumor ENO1 expression and patients’ survival using Kaplan-Meier analysis with the log-rank test. In the 136 glioma cases with survival data, we observed that the level of ENO1 protein expression was significantly correlated with overall survival. Patients with tumors expressing low levels of ENO1 expression had better survival than those with tumors expressing high expression (Figure 2) (*P* < 0.001).

### Stably downregulated ENO1 expression suppresses cell proliferation, colony formation and in vivo tumorigenicity

**Figure 3:**
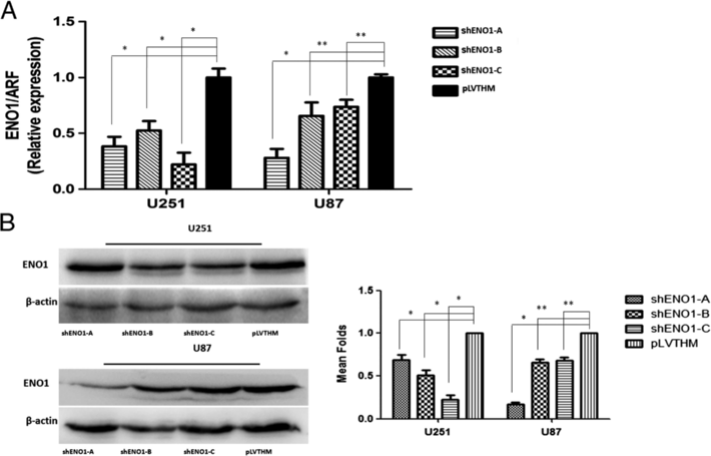
**Effect of shRNA to stably knock down the expression of ENO1 in human glioma cell lines U251 and U87.** Different treatments included PLV-Ctr. **(A)**. RT-PCR shows transcriptional levels of the ENO1 gene with ARF used as a loading control. **(B)**. Western blot showing protein expression levels in shENO1 and PLV-Ctr treatments. A representative image of three different experiments is shown. β-actin served as a loading control. Bar graph shows the relative expression of protein among the groups. Data are presented as mean ± SD for three independent experiments (*P < 0.05, **P > 0.05).

We used a lentiviral shRNA vector to specifically and stably knock down the expression of ENO1 in U87 and U251 cell lines that were established from high-grade tumors. Transcriptional levels of ENO1 were assessed by RT-PCR, with the most efficient knockdowns from shENO1-C in U251 cell line and shENO1-A in U87 cell line compared to the empty vector controls [pLVTHM-GFP-Control (PLV-Ctr)] (*P* < 0.01) (Figure 3A). Consistent results for protein levels were observed by Western blot (Figure 3B).

**Figure 4:**
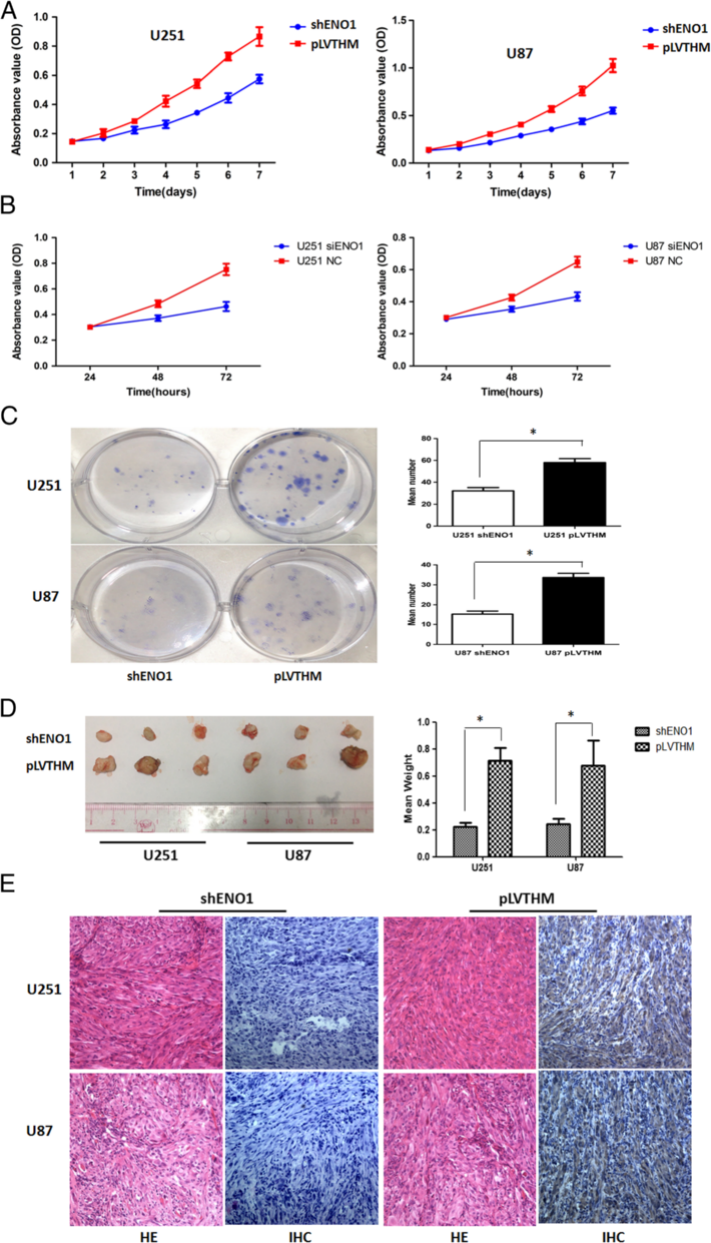
**Stably downregulated ENO1 expression suppressed cell proliferation in vitro and tumorigenicity in vivo. (A)**. Effect of ENO1 knockdown on U251 and U87 cell proliferation as measured by MTT assay. Absorbance was read at 490 nm with averages from triplicate wells. Data are presented as mean ± SD for three independent experiments. **(B)**. Transiently reducing the expression of ENO1 by siRNA inhibited cell proliferation in glioma U251 and U87 cells. **(C)**. In vitro proliferative ability of glioma cells was significantly decreased in ENO1-suppressed cells compared to PLV-Ctr cells by colony formation assay. **(D)**. When compared with PLV-Ctr, tumorigenicity of shENO1-U25 and shENO1-U87 cells was markedly reduced in vivo (*P < 0.05). **(E)**. Immunohistochemical (IHC) staining of ENO1 expression in subcutaneous tumors of mice injected with shENO1 and PLV-Ctr cells.

Subsequently, we examined the effect of decreased ENO1 expression on glioma cell growth in vitro. Using an MTT assay, we found that the growth of shENO1 U251 and U87 cells was significantly slower than the PLV-Ctr cells from day 1 (*P* < 0.05) (Figure 4A). Interestingly, similar results were also observed in siRNA-mediated suppression of ENO1 in glioma cells. We found that knocking down endogenous ENO1 expression decreased cell proliferation compared to the negative control (NC) groups (Figure 4B). Colony formation assay showed that suppressing ENO1 significantly inhibited cell proliferation compared to PLV-Ctr cells (Figure 4C). To confirm the growth enhancing effects of ENO1, we performed an in vivo tumorigenesis study by inoculating shENO1 U251 and U87 cells into nude mice. Mice in the shENO1-U251 and PLV-Ctr groups were sacrificed 18 days after inoculation, with average tumor weights of 0.223 g and 0.713 g, respectively (*P* < 0.01). In shENO1-U87 and PLV-Ctr groups, the average tumor weights were 0.243 g and 0.677 g, respectively (*P* < 0.01) (Figure 4D). Immunohistochemistry staining verified normal expression of ENO1 in the PLV-Ctr–xenografted tumors compared with reduced or lack of expression in shENO1–xenografted tumors (Figure 4E). These results suggested a significant inhibitory effect of decreased ENO1 on in vivo tumorigenesis.

### Knockdown of ENO1 suppresses glioma cell migration and invasion in vitro

**Figure 5:**
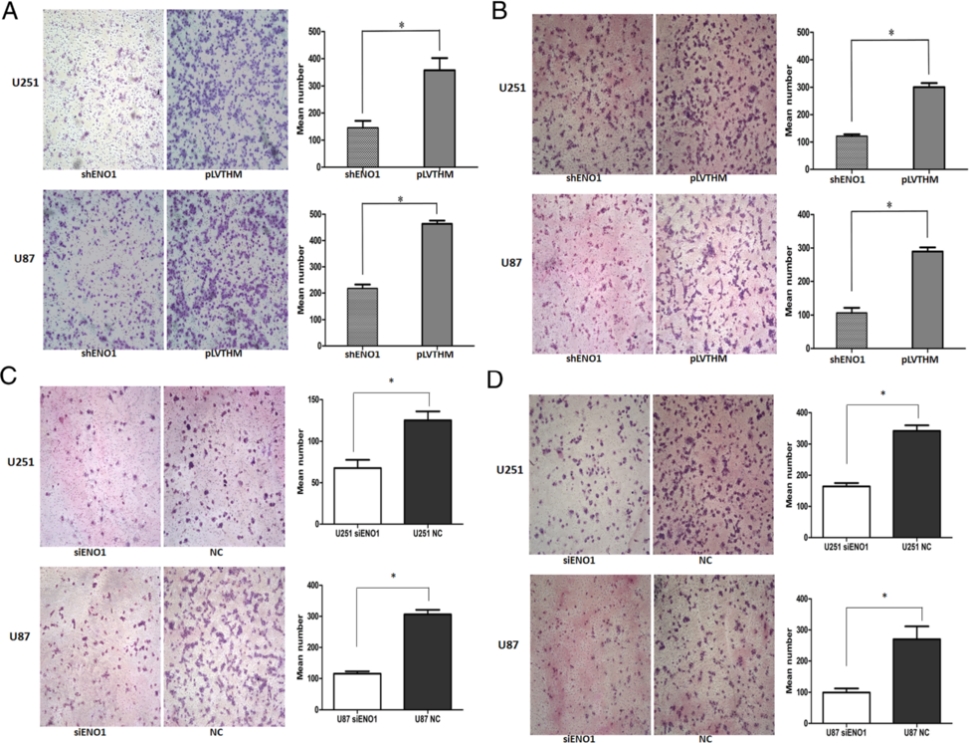
**Stably inhibited ENO1 expression decreases cell migration and invasion. (A)**. Stably downregulating ENO reduced the migration ability of shENO1-U251 and shENO1-U87 cells in vitro. **(B)**. Stably suppressed ENO1 reduced in vitro invasion of shENO1-U251 and shENO1-U87 cells. **(C)**. Transiently downregulated ENO1 dramatically decreased the migration ability of U251 and U87 cells in vitro. **(D)**. Transiently suppressed ENO1 inhibited in vitro invasion of U251 and U87 cells. Data were presented were presented as mean ± SD for three independent experiments. *P < 0.05, statistically significant difference.

To examine the effect of ENO1 on cell migration, shRNA-ENO1 infected U251 and U87 glioma cells were cultured on Transwell apparatus. After 12 hr incubation, the percentage of migrated cells in both shENO1-U251 and shENO1-U87 glioma cell groups was significantly less than that in the PLV-Ctr cells (for both *P* < 0.01) (Figure 5A). Using a Boyden chamber coated with matrigel, we determined changes in cell invasion after 16 hr incubation. Compared with the PLV-Ctr cells, shRNA-ENO1 U251 and U87 glioma cells both showed significantly decreased invasion (*P* < 0.01 for each) (Figure 5B). Similar to the stably suppressed ENO1 expression results, downregulation of ENO1 using siRNA-ENO1 also inhibited cell migration and invasion in U251 and U87 cells (Figure 5C, D).

### ENO1 controls the expression of cell cycle and EMT associated genes in glioma

**Figure 6:**
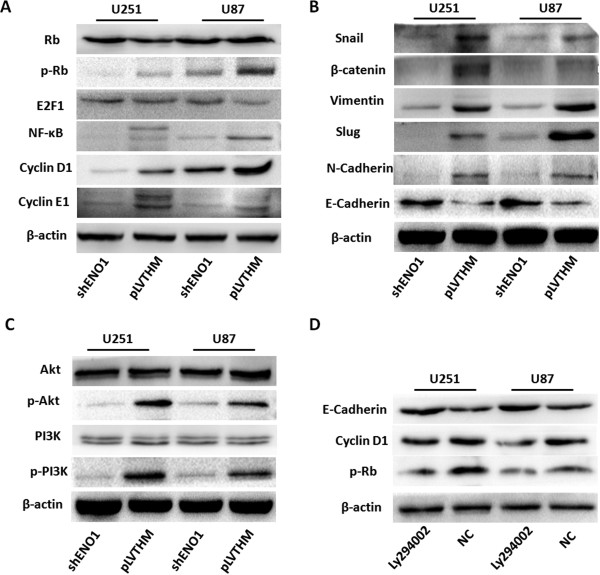
**ENO1 controls the expression of cell cycle and EMT-associated genes in glioma via PI3K/Akt pathway. (A)**. Knocking down endogenous ENO1 expression reduced the expression of pRb (Ser 780), NF-κB, and oncogenic cell cycle regulators including Cyclin D1 and Cyclin E1. However, total Rb and E2F1 were not affected. **(B)**. Suppressing ENO1 expression decreased the expression of EMT-marker genes including Snail, β-catenin, Vimentin, Slug and N-cadherin but enhanced E-cadherin expression. **(C)**. Reduced ENO1 expression depressed the expression of phos-PI3K, and Akt, but not their total protein levels. **(D)**. Western blot analyses of E-cadherin, Cyclin D1, p-Rb in glioma U251 and U87 cells after LY294002 treatment. Each experiment was repeated three times.

To further study the mechanism by which ENO1 regulates cell proliferation, migration and invasion, we examined protein levels of cell cycle and EMT-associated genes in glioma U251 and U87 cells with stably suppressed ENO1 expression. Knocking down endogenous ENO1 expression inhibited the activation of pRb (Ser 780), NF-κB and oncogenic cell cycle regulators including Cyclin D1 and Cyclin E1. The expression of total Rb and E2F1 were not affected (Figure 6A). Further, we found that suppressing ENO1 expression decreased the expression of Snail, β-catenin, Vimentin, Slug and N-cadherin, while elevated E-cadherin expression (Figure 6B).

### ENO1 regulates PI3K/Akt pathway

The PI3K/Akt protein complex has been reported to play an important role in modulating cell cycle and EMT activities. We examined the effect of ENO1 on PI3K/Akt pathway and found that reduced ENO1 significantly decreased the phosphorylation of PI3K and Akt, but not their total protein levels (Figure 6C). Treatment of glioma U251 and U87 cells with LY294002 had a similar effect on E-Cadherin, Cyclin D1, and p-Rb as ENO1 knockdown (Figure 6D). These results suggested that ENO1 is an upstream factor modulating the PI3K/Akt pathway in glioma.

## Discussion

Gliomas cells are known to have more glycolytic activities than NB tissue, especially in glioblastomas (GBM) [15]. Robust migration of GBM cells has been previously demonstrated under glycolytic conditions and their pseudopodia contain increased glycolytic and decreased mitochondrial enzymes [16]. GBM commonly exhibit large areas of hypoxia in situ, with the potential for accumulation of intracellular metabolic acids, such as lactic and citric acid [17]. ENO1 is a glycolysis enzyme crucial for anabolic processes and energy generation [18], and is the major enolase isoform in GBM, accounting for 75–90% of cellular enolase activity [19]. Silencing of ENO2 mediated by shRNA selectively inhibits growth, survival and the tumorigenic potential of ENO1-deleted GBM cells, and that the enolase inhibitor phosphonoacetohydroxamate is selectively toxic to ENO1-deleted GBM cells relative to ENO1-intact GBM cells or normal astrocytes [20]. Enolase was the most sensitive marker of pathological change and was the only enzyme found to be elevated in the cerebrospinal fluid of patients with low grade astrocytomas [21]. Therefore, enzymes and other proteins that protect glycolytic activity from acidosis need to be identified and investigated as targets to improve therapies of these invasive tumors.

Since the ENO1 promoter contains a hypoxia responsive element, the overexpression of ENO1 is associated with tumor development through a process known as aerobic glycolysis or the Warburg effect [22]. The mechanism of the Warburg effect was uncertain until the recent identification of upregulation of glycolytic enzymes by hypoxia-inducible factor. Together these findings suggest that ENO1 may play a unique role in the Warburg effect of glioma cells. Nevertheless, more detailed and non-overlapping potential functions of the protein remain poorly understood. Several studies have shown that, besides its major role in glycolysis, ENO1 is a multifunctional protein displaying a range of distinct activities. It is a hypoxic stress protein in endothelial cells [23], a heat shock protein in yeast, a lens crystalline and an autoimmune antigen [24].

Though Enolase was observed increased in the cerebrospinal fluid of patients with low grade astrocytoma [21], in the present study, we investigated tumor tissues. First, we confirmed that ENO1 mRNA levels were higher in 45 glioma samples than in 15 NB tissues. Consistent with mRNA levels, ENO1 protein expression was significantly elevated in 10 glioma samples compared to 4 NB tissues. We used immunohistochemistry to further examine the expression level and subcellular localization of ENO1 in glioma and NB tissues. We found that ENO1 was mainly localized in the cytoplasm of glioma tissues while weakly expressed in cytoplasm in NB tissues. We also observed that the expression level of ENO1 was positively correlated with tumor grading as there was a significant difference between high and low grade gliomas. Multivariate analyses showed that increased expression of ENO1 protein was a significant predictor of poor prognosis for glioma patients. These data strongly imply an oncogenic role for ENO1 in glioma tumorigenesis.

In previous studies, elevated expression of ENO1 was positively associated with progression and poor prognosis in neuroendocrine tumors, neuroblastoma, pancreatic cancer, prostate cancer, cholangiocarcinoma, thyroid carcinoma, lung cancer, hepatocellular carcinoma and breast cancer, and suggested an involvement of ENO1 in tumor progression [25-32]. The biological functions of ENO1 found in this study provided a mechanistic basis for the pathological and clinical observations. We found that stably decreased expression of ENO1 by shRNA inhibited glioma cell proliferation, migration, and invasion in vitro and decreased tumorigenesis in vivo compared to the PLVM-Ctr groups.

Cell cycle progression is partly dependent on the tight complex network of Cyclin D, CDKs, Rb and Rb-related proteins [33]. Both of these pathways are required for hyperphosphorylation of the retinoblastoma gene product (pRb) [34]. In this investigation, we examined key cell growth regulators and observed that Cyclin D1, Cyclin E1, pRb, and NF-κB were downregulated after stable ENO1 knockdown in glioma U251 and U87 cells.

EMT is involved in cell migration and invasion which are key steps in the progression of glioma [35]. The loss of E-cadherin and overexpression of mesenchymal cell markers such as N-cadherin, Vimentin are hallmarks of EMT [36]. Our data indicated that knockdown of ENO1 resulted in restoration of E-cadherin expression and suppression of Vimentin expression in glioma cells. Furthermore, we observed that ENO1 downregulation also inhibited the expression of other mesenchymal cell markers such as Snail, N-Cadherin, β-Catenin and Slug.

PI3K/Akt is a classical signal pathway [37-39], and its activated status induces cell growth [40,41], increases the expression of Snail and promotes the EMT [42]. In this study, we also observed that decreased ENO1 expression suppressed pPI3K and pAkt levels, while not affecting total PI3K and Akt protein levels. Suppression of PI3K in U251 and U87 glioma cells with LY294002 treatment led to the upregulation of E-cadherin while decreasing Cyclin D1 and p-Rb. We hypothesize that ENO1 may contribute to tumor progression via the PI3K/Akt pathway mediating the activity of Cyclin D1, Cyclin E, and NF-κB, which will eventually result in hyperphosphorylation of Rb and downregulation of E-cadherin mediated classical EMT.

## Conclusion

In summary, ENO1 expression may have significant value as an unfavorable progression indicator for glioma patients. We provide compelling evidence that attenuated ENO1 expression leads to suppressed cell growth, migration and invasion progression by inactivating the PI3K/Akt pathway in glioma cells.

## Materials and methods

### Cell culture and sample collection

The human glioma cell line U251,U87 were purchased from the Chinese Academy of Sciences (Shanghai, China) and grown in Dulbecco’s modified Eagle’s medium (DMEM) (Hyclone, Logan, UT) supplemented with 10% fetal calf serum (ExCell, Shanghai, China). All cell lines were cultured at 37°C in a humidified atmosphere of 5% CO_2_. A total of 136 paraffin-embedded glioma and 15 NB tissues samples were obtained from the Nanfang Hospital of Southern Medical University, Guangzhou, China. These cases were from 88 males and 48 females with age ranging from 11 to 68 years (median, 41.7 years). For the use of these clinical materials for research purposes, prior consent from patients and approval from the Ethics Committees of Nanfang Hospital were obtained. All specimens had confirmed pathological diagnosis and were classified according to the World Health Organization (WHO) criteria.

### RNA isolation, reverse transcription, and qRT-PCR

RNA was extracted from the U87 and U251 cell lines, glioma tissues and NB tissues using Trizol (Takara, Shiga, Japan). For ENO1, RNA was transcribed into cDNA and amplified with specific sense: 5′- GCCGGCTTTACGTTCACCTC-3′, antisense primer: 5′- GTTGAAGCACCACTGGGCAC-3′. ARF gene was used as an internal control using the sense primer 5′-ATCTGTTTCACAGTCTGGGACG-3′ and antisense primer 5′-CCTGCTTGTTGGCAAATACC-3′. The assays were performed in accordance with manufacturer’s instructions (Takara, Shiga, Japan). Cycling conditions were 95°C for 10 min to activate DNA polymerase, followed by 45 cycles of 95°C for 15 s, 55°C for 15 s, and 72°C for 10s. Specificity of amplification products was confirmed by melting curve analysis. PCR reactions for each gene were repeated three times. Independent experiments were done in triplicate.

### Immunohistochemistry

Paraffin sections (4 μm) from samples were deparaffinized in 100% xylene and re-hydrated in descending ethanol series and water according to standard protocols. Heat-induced antigen retrieval was performed in 10 mM citrate buffer for 2 min at 100°C. Endogenous peroxidase activity and non-specific antigens were blocked with peroxidase blocking reagent containing 3% hydrogen peroxide and serum, followed by incubation with rabbit anti-human ENO1 antibody (1: 150) (48 kDa, Proteintech, USA) overnight at 4°C. After washing, the sections were incubated with biotin-labeled rabbit anti-goat antibody for 10 min at room temperature, and subsequently were incubated with streptavidin-conjugated horseradish peroxidase (HRP) (Maixin, Fuzhou, China). The peroxidase reaction was developed using 3,3-diaminobenzidine (DAB) chromogen solution in DAB buffer substrate. Sections were visualized with DAB and counterstained with hematoxylin, mounted in neutral gum, and analyzed using a bright field microscope.

### Evaluation of staining

The immunohistochemically stained tissue sections were reviewed and scored separately by two pathologists blinded to the clinical parameters. Expression of ENO1 in the nucleus and in the cytoplasm was independently evaluated. For cytoplasmic staining, the score was evaluated according to the sum of cytoplasm staining intensity and the percentage of positive staining areas of cells. The staining intensity was scored as previously described (0–3) [43,44] and the percentage of positive staining areas of cells was defined as a scale of 0–3 (0: <10%, 1: 10-25%, 2: 26-75%, and 3: >76%). For nuclear staining, the staining score was defined based on the sum of nuclear staining intensity and the number of positive nuclear staining. Nuclear staining intensity score was consistent with cytoplasm. The positive nuclear staining scores were defined as follows: 0: <20%, 1: 20-49%, 2: 50-79%, and 3: >80%. The sum of the cytoplasm and nuclear staining scores were used as the final staining score for ENO1 (0–12). For statistical analysis, a final staining score of 0–4 and 5–6 in cytoplasm or 0–3 and 4–6 in nucleus was considered to be low or high expression, respectively.

### Western blot analysis

Western blot was carried out according as described [45] with rabbit polyclonal anti-ENO1 antibody (1:1000; Proteintech, USA), anti-NF-κB, Cyclin D1, Cyclin E1 and E2F1 antibody (1:400; Santa Cruz Biotechnology, Santa Cruz, USA), anti-Rb, pRb (Ser,780), Akt, pAkt (Ser473), PI3K, pPI3K (Tyr458), Snail, Slug, β-catenin, E-Cadherin, N-Cadherin and Vimentin antibody (1:1000; Cell Signaling Technology, Danvers, USA). An HRP-conjugated anti-rabbit IgG antibody was used as the secondary antibody (Zhongshan, Beijing, China). Signals were detected using enhanced chemiluminescence reagents (Pierce, Rockford, IL).

### Establishment of glioma cell line with stable expression of ENO1 short hairpin RNA

The preparation of lentiviruses expressing human ENO1 short hairpin RNA (shRNA-9449,9450,9452) (ENO1-1,sense: 5′-CCGGAATGTCATCAAGGAGAAATATCTCGAGATATTTCTCCTTGATGACATTTTTTTG-3′, antisense: 5′-AATTCAAAAAAATGTCATCAAGGAGAAATATCTCGAGATATTTCTCCTTGATGACATT-3′; ENO1-2, sense: 5′-CCGGCGTGAACGAGAAGTCCTGCAACTCGAGTTGCAGGACTTCTCGTTCACGTTTTG-3′, antisense: 5′-AATTCAAAACGTGAACGAGAAGTCCTGCAACTCGAGTTGCAGGACTTCTCGTTCACG-3′; ENO1-3, sense: 5′-CCGGCCACTGTTGAGGTTGATCTCTCTCGAGAGAGATCAACCTCAACAGTGGTTTTTG-3′, antisense: 5′-AATTCAAAAACCACTGTTGAGGTTGATCTCTCTCGAGAGAGATCAACCTCAACAGTGG-3′) were performed using the pLVTHM-GFP lentiviral RNAi expression system [46]. U87 and U251 cells were infected with lentiviral particles containing specific or negative control vectors, and polyclonal cells with GFP signals were selected for further experiments using FACS flow cytometry. Total RNA of these cell clones was isolated, and levels of ENO1 mRNA were measured using real-time PCR analysis.

### Transient transfection with siRNAs

Small-interfering RNA (siRNA) for ENO1 was designed and synthesized by Guangzhou RiboBio (RiboBio Inc, China). Three siRNAs targeting on ENO1 gene were designed and synthesised, the most effective siRNA (siENOA) identified by Real Time-PCR was applied for the further experiments. The sequence of siENO1 is: sense: 5′-GCAUUGGAGCAGAGGUUUAdTdT-3′; anti-sense: 3′-dTdTCGUAACCUCGUCUCCAAAU-5′. The sequence of si-negative control (si-Ctr) was also designed by RiboBio (RiboBio Inc., China). Twenty-four hours prior to transfection, glioma cells U87 and U251 were plated onto a 6-well plate or a 96-well plate (Nest Biotech, China) at 30–50% confluence. They were then transfected into cells using TurboFect TM siRNA Transfection Reagent (Fermentas, Vilnius, Lithuania) according to the manufacturer’s protocol. Cells were collected after 48–72 hr for further experiments.

### Cell viability and proliferation assay

Cell proliferation was analyzed using MTT assay. Cells were seeded in 96-well plates at a density of 700 cells/well. The cells were incubated for 1, 2 or 3 days. Twenty microliters of MTT (5 mg/ml) (Sigma, St. Louis, MO) was added to each well and incubated for 4 hr. At the end of incubation, supernatants were removed, and 150 μl of DMSO (dimethyl sulfoxide) (Sigma, St. Louis, MO) was added to each well. The absorbance value (OD) of each well was measured at 490 nm. For each experimental condition, eight wells were used. Experiments were performed in triplicate.

### Cell migration and invasion assays

In vitro cell migration and invasion assays were examined according to our previous study [47]. For the cell migration assay, 1 × 10^4^ cells in 100 μl DMEM medium without FCS were seeded on a fibronectin coated polycarbonate membrane insert in a Transwell apparatus (Costar, MA). In the lower chamber, 600 μl DMEM with 10% FCS was added as chemoattractant. After the cells were incubated for 6 hr at 37°C in a 5% CO_2_ atmosphere, the insert was washed with PBS, and cells on the top surface of the insert were removed with a cotton swab. Cells adhering to the lower surface were fixed with methanol, stained with Giemsa solution and counted under a microscope in five predetermined fields (200×). All assays were independently repeated at least three times. For the cell invasion assay, the procedure was similar to the cell migration assay, except that the Transwell membranes were precoated with 24 μg/μl Matrigel (R&D Systems, USA) and the cells were incubated for 6 hr at 37°C in a 5% CO2 atmosphere. Cells adhering to the lower surface were counted the same way as the cell migration assay.

### Colony formation assay

Cells were plated in 6-well culture plates at 100 cells/well. Each cell group had 2 wells. After incubation for 12 days at 37°C, cells were washed twice with PBS and stained with Giemsa solution. The number of colonies containing >50 cells was counted under a microscope. The colony formation efficiency was calculated as (number of colonies/number of cells inoculated) × 100%.

### In vivo tumorigenesis in nude mice

A total of 1 × 10^6^ logarithmically growing U251 cells transfected with pLVTHM-GFP -ENO1 and the control pLVTHM-GFP vector (N = 6 per group) in 0.1 ml DMEM medium were subcutaneously injected into the left-right symmetric flank of 4-6-week-old male BALB/c nu/nu mice. The mice were maintained in a barrier facility on HEPA-filtered racks. The animals were fed an autoclaved laboratory rodent diet. All animal studies were conducted in accordance with the principles and procedures outlined in the National Institutes of Health Guide for the Care and Use of Animals under assurance number A3873-1. After 18 days, the mice were sacrificed respectively and tumor tissues excised and weighed.

### Statistical analysis

All quantified data represented an average of at least triplicate samples. SPSS 13.0 and Graph Pad Prism 4.0 software were used for statistical analysis. Data are presented as mean ± SD. One-way ANOVA or two-tailed Student’s t-test was used for comparisons between groups. Chi-square test or Fischer’s were used to identify differences between categorical variables. Survival analysis was performed using Kaplan-Meier method. Multivariate Cox proportional hazards method was used for analyzing the relationship between the variables and patient’s survival time. Differences were considered statistically significant when *P* < 0.05.

## Competing interests

The authors have declared that no competing interests exist.

## Authors’ contributions

STQ, YS, ZL and WYF conceived and designed the experiments. STQ, YS, QSL, ZH, WYF, ZL, HL, TSQ, XAZ, ZYL and YL performed all the experiments. STQ, YS, QSL, WYF and ZL analyzed the data. STQ, YS, ZL, WYF provided material and collected the clinical data. STQ, YS, QSL, ZL and WYF were involved in writing the paper. All authors gave final approval for the manuscript to be submitted for publication.
