# Erratum to: Alpha-enolase as a potential cancer prognostic marker promotes cell growth, migration, and invasion in glioma

**DOI:** 10.1186/1476-4598-13-235

**Published:** 2015-01-20

**Authors:** Ye Song, Qisheng Luo, Hao Long, Zheng Hu, Tianshi Que, Xi’an Zhang, Zhiyong Li, Gang Wang, Liu Yi, Zhen Liu, WeiYi Fang, Songtao Qi

**Affiliations:** Department of Neurosurgery, Nanfang Hospital, Southern Medical University, Guangzhou, Guangdong PR China; Cancer Research Institute of Southern Medical University, Guangzhou, Guangdong PR China; Department of Neurosurgery, Affiliated Hospital, Youjiang Medical College for Nationalities, Baise, Guangxi PR China; Department of Pathology, Basic School of Guangzhou Medical College, Guangzhou, Guangdong PR China

## Correction

After the publication of this work [1] it was brought to the authors’ attention that the U251-pLVTHM panel in Figure fiveB and the U251 negative control (NC) panel in Figure fiveD contained a duplication in error. The correct version of Figure five (Figure 1 here) is given below.

**Figure 1:**
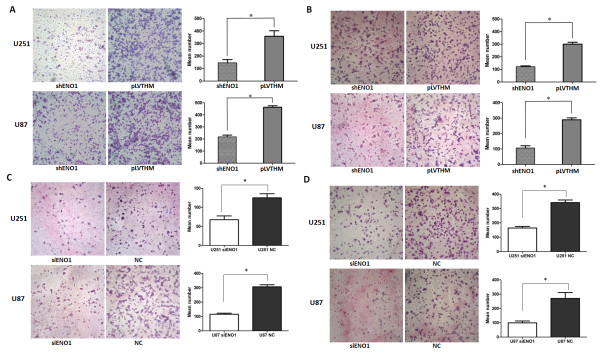
**Stably inhibited ENO1 expression decreases cell migration and invasion.** **(A)**. Stablydownregulating ENO reduced the migration ability of shENO1-U251 and shENO1-U87 cells in vitro. **(B)**. Stably suppressed ENO1 reduced in vitro invasion of shENO1-U251 and shENO1-U87 cells. **(C)**. Transiently downregulated ENO1 dramatically decreased the migration ability of U251 and U87 cells in vitro. **(D)**. Transiently suppressed ENO1 inhibited in vitro invasion of U251 and U87 cells. Data were presented were presented as mean ± SD for three independent experiments. *P < 0.05, statistically significant difference.

The authors regret any inconvenience that this inaccuracy may have caused.
